# Mortality Attributable to *Clostridioides difficile* Infection: The Rising Burden of Disease in European Countries

**DOI:** 10.3390/medicina60081222

**Published:** 2024-07-28

**Authors:** Irena Ilic, Ivana Zivanovic Macuzic, Milena Ilic

**Affiliations:** 1 Faculty of Medicine, University of Belgrade, 11000 Belgrade, Serbia; 2 Department of Anatomy, Faculty of Medical Sciences, University of Kragujevac, 34000 Kragujevac, Serbia; 3 Department of Epidemiology, Faculty of Medical Sciences, University of Kragujevac, 34000 Kragujevac, Serbia

**Keywords:** *Clostridium difficile*, epidemiology, mortality, trends

## Abstract

*Background and Objectives*: *Clostridioides difficile* infection is a major public health issue, being among the main causes of mortality due to healthcare-associated diarrhea. This study aimed to assess the trends in mortality attributable to *Clostridioides difficile* infections in European countries over a period of 30 years. *Materials and Methods*: A descriptive epidemiological study was conducted, with the application of an ecological study design, to evaluate the trends in mortality due to *Clostridioides difficile* infection in the Central, Eastern, and Western European sub-regions from 1990 to 2019. The Global Burden of Disease study database was used. Trends were evaluated with the joinpoint regression analysis. *Results*: In both sexes, about 76% of all deaths attributable to *Clostridioides difficile* infections were recorded in the Western European sub-region in 2019. The age-standardized rates of the burden of *Clostridioides difficile* infection in 2019 were the highest in the Central European sub-region, followed by the Western European sub-region, while the lowest rates were observed in the Eastern European sub-region. A significantly increasing trend in mortality attributable to *Clostridioides difficile* infection from 1990 to 2019 was recorded both in males (by +2.1% per year) and females (by +2.8% per year). The burden of *Clostridioides difficile* infection showed increasing trends in most of the European countries, significantly correlating with the country’s development, according to the Human Development Index. *Conclusions*: The rising burden of *Clostridioides difficile* infection in European countries in the last few decades suggests a need for improving public health measures, with a focus both on the hospital setting and community.

## 1. Introduction

*Clostridioides difficile* (*C. difficile*, formerly termed *Clostridium difficile*) is a ubiquitous human pathogen which poses a major global health challenge worldwide, because it is considered the leading cause of healthcare-associated colitis [1]. The mortality rates from *Clostridioides difficile* show a significant variation worldwide. In the United States, over the 2010–2019 period, the mortality rate decreased from 3.2% to 1.4%, with females showing higher mortality rates in hospitalizations with *Clostridioides difficile* infections [2]. The estimated rates of in-hospital deaths from healthcare-associated *Clostridioides difficile* infections not-significantly changed from 8.3 per 100,000 persons in 2011 to 5.0 per 100,000 persons in 2017 in the United States [3]. Similarly, a study in Scotland showed decreasing trends in mortality following *Clostridioides difficile* infection over the 2010–2016 period, from 20.5% to 15.6% [4]. Still, there are data showing that the *Clostridioides difficile*-attributable mortality was 1.5% before 2000 and increased up to 5.7% since then in the endemic periods and up to 16.7% in epidemic periods in the United States [5]. Nevertheless, the impact of the COVID-19 pandemic remains yet to be determined, given that there was a notable increase in *Clostridioides difficile* infections in oncological patients in Spain [6]. Despite the recently observed declines in incidence of *Clostridioides difficile* infections in some European countries, this disease still shows substantial associated morbidity, mortality, and costs [7]. A large systematic review that examined reports from Europe found that the mortality from *Clostridioides difficile* at 30 days was the lowest in France (2%) and the highest in the United Kingdom (42%), with reports of in-hospital *Clostridioides difficile* mortality ranging from 0% in Latvia and 3.5% in Belgium to 37% in the United Kingdom and 44.4% in Austria [8]. It is particularly worrisome that the mortality rates in Europe have shown a two-fold increase over the 1999–2004 period [8]. In addition, substantial economic costs are associated with *Clostridioides difficile* infections, with about USD 800 million of annual costs per year in the United States and an estimated EUR 300 million in the European Union [9], with a large systematic review of available *Clostridioides difficile*-associated cost reports showing an increasing economic burden of *Clostridioides difficile* infections in Europe [8]. 

Healthcare-associated *Clostridioides difficile* infections are closely related to the use and misuse of antibiotics, particularly those of a broad spectrum [10]. Contributing factors also include a pre-existing disease and an increasing age, which can compromise the host’s immune response. The use of antibiotics can cause changes in the gut microbiome, leading to dysbiosis in the large intestine and facilitating *Clostridioides difficile* colonization of the human gastrointestinal tract [11]. Up to 5% of healthy individuals are considered to be asymptomatic carriers of *Clostridioides difficile* [12]. This infection is primarily transmitted via the fecal–oral route, but it can also be transmitted from the environment. In hospitals, *Clostridioides difficile* spores are most often transmitted through contaminated surfaces, thus underlining the importance of infection prevention and control measures, alongside antibiotic stewardship programs [13]. Once the spores of anaerobic *Clostridioides difficile* reach the gut of susceptible individuals, they germinate and produce toxins leading to either an asymptomatic carriage or abdominal cramping and pain with diarrhea of mild to severe intensity. In the most severe cases, this infection leads to pseudomembranous colitis, toxic megacolon, or perforation and death [11,13].

Recent research has indicated an increase in *Clostridioides difficile* infections in communities and in nursing homes outside of hospital settings or healthcare facilities and without a history of recent antibiotic use in these individuals, underlining the threat that this agent poses to public health [14]. Over the 2011–2017 period, the estimated in-hospital death rates of persons who acquired a *Clostridioides difficile* infection outside of hospital settings or healthcare interventions showed almost no significant change from 1.6 deaths per 100,000 persons in 2011 to 1.3 deaths per 100,000 persons in 2017 [3]. The increasing burden of community-associated infections and the reported finding that in 2017 they represented 50% of all *Clostridioides difficile* cases in the United States [3] point to the need for further research into the diverse epidemiology of *Clostridioides difficile* infection.

A recent meta-analysis of the global widespread *Clostridioides difficile* burden has identified evidence gaps and has reported that for community-associated *Clostridioides difficile* infections, the highest cumulative incidence rates in all age groups were recorded in Europe, while the recorded estimates for healthcare-associated *Clostridioides difficile* infection rates in Europe were between 3.14 and 4.08 per 10,000 patient days [15]. According to a systematic review of available mortality estimates in Europe, there is a knowledge gap, in particular for Eastern and Southern Europe [8]. There is also a paucity of published comprehensive reports on *Clostridioides difficile* mortality trends in Europe [7]. The transmissibility and significant morbidity and mortality associated with *Clostridioides difficile* infections highlight the need for improved infection-prevention measures, such as restrictive antibiotic stewardship programs, which can both prevent infections and also reduce the occurrence of multi-drug-resistant pathogens [16].

The changing epidemiology of *Clostridioides difficile* infection emphasizes the need for comprehensive and up-to-date mortality estimates of *Clostridioides difficile* in Europe, in order to provide an insight into the implementation of existing infection-control protocols and to enable improvements in infection prevention, detection, surveillance, and aid in mitigating the mortality associated with this infection through targeted public health interventions and resource allocations. Therefore, investigating disease frequency and temporal trends is crucial in its prevention. This study aimed to assess the trends in mortality attributable to *Clostridioides difficile* infections in European countries over a period of 30 years.

## 2. Materials and Methods

### 2.1. Study Design

A descriptive epidemiological study (using an ecological study design) was conducted to evaluate the regional and national trends in mortality due to *Clostridioides difficile* infections in the Central, Eastern, and Western European sub-regions over the 1990–2019 period.

### 2.2. Data Source

The Global Burden of Disease (GBD) 2019 study database was used to obtain data on mortality due to *Clostridioides difficile* infections [17]. The GBD 2019 study provides systematically and comprehensively collected and analyzed data on mortality and disability, with rigorous and comparable estimates of many health problems worldwide, based on a combination of numerous relevant sources of data (i.e., vital statistics, verbal autopsies, surveillance data, hospital records, household surveys, censuses, registries, use of health services, medical insurance, disease notifications, and others) to estimate the burden of disease [17]. The GBD database estimates the number of deaths due to *Clostridioides difficile* infections at the country–year level, while death rates were calculated using the Cause of Death Ensemble model. The GBD study adheres to the Guidelines for Accurate and Transparent Health Estimates Reporting (GATHER) [18].

Also, the GBD produces comprehensive and internally consistent estimates of populations by sex and age for each calendar year since 1950 at the national level [19]. Annual and single-year age estimates of the population for each country were generated using a Bayesian hierarchical cohort component model that analyzed estimated age-specific fertility and mortality rates, along with censuses and population registry years. Only population estimates from the GBD are compliant with the GATHER [18].

In this study, the GBD estimates of mortality attributed to *Clostridioides difficile* infection as the underlying cause of death are presented [17]. According to the World Health Organization, the underlying cause of death is defined as ‘the disease or injury which initiated the train of morbid events leading directly to death, or the circumstances of the accident or violence which produced the fatal injury’ [20]. For *Clostridioides difficile* infections, the GBD cause list covered code A04.7 (“Enterocolitis due to *Clostridium difficile*”, based on the 10th revision of the International Classification of Diseases) and code 008.45 (“Intestinal infection due to *Clostridium difficile*”, based on the 9th revision of the International Classification of Diseases).

Mortality due to *Clostridioides difficile* infection was presented within the three European sub-regions (Western, Central, and Eastern). Also, mortality due to *Clostridioides difficile* infection was presented for 44 countries in Europe, comprising about 745 million inhabitants in 2019 [17].

### 2.3. Measures

This analysis included estimates of mortality and years of life lost (YLLs) due to *Clostridioides difficile* infections in countries in Europe [17].

Age-standardized rates (ASRs) were used to assess the trends in burden attributable to *Clostridioides difficile* infections in European countries over the 1990–2019 period. The ASRs per 100,000 persons were estimated via the direct standardization method, with the world standard population developed for the GBD study [17]. For countries (such as Andorra, Monaco, and San Marino) that had less than 100,000 inhabitants, the data are shown but not taken into account in the comparison due to the instability of the rates.

Further, trends in age-specific and sex-specific rates in burden attributable to *Clostridioides difficile* infections are shown. In addition, subgroup analyses were conducted, with the age groups divided into 20 strata (<5/5–9/10–14/15–19/20–24/ …/95+ years) for males and females separately.

Years of life lost (YLLs) refers to the data on the total years of life lost due to the premature mortality of the population [17]. As a summary measure of the total premature mortality attributed to *Clostridioides difficile* infection as the cause of death, YLLs is an indicator of the effect of that premature mortality on the population. One YLL represents a loss of one year of life.

The Human Development Index (HDI) is a composite estimate of the level of a country’s development, representing the country’s achievements in three basic dimensions: health (estimated via life expectancy at birth), knowledge (estimated via mean years of schooling for adults ≥ 25 years old), and standard of living (estimated via gross national income per capita) [21].

### 2.4. Statistical Analysis

To estimate the magnitude and direction of the trends in mortality over time, a joinpoint regression analysis was performed (using the Joinpoint regression software, Version 4.9.0.0—March 2021, available through the Surveillance Research Program of the US National Cancer Institute), as proposed by Kim and coauthors [22]. This program rigorously examined, identified, and quantitatively characterized significant change points within the time-series data concerning the burden of *Clostridioides difficile* infections across European countries. The joinpoint regression analysis calculates the Annual Percentage Change (APC) in the temporal trends in mortality and YLLs, as well as the Average Annual Percentage Change (AAPC) for the entire observed period, along with corresponding 95% confidence intervals (95% CIs) [23]. The maximum number of joinpoints allowed was 5 (with six lines), while zero joinpoints corresponded to a straight line, and this analysis was conducted with the default mode of the software—the Grid Search method. The Monte Carlo method of 4499 permutation tests was used to choose the optimal fitting model. When a change in the trend was significant (according to the statistical significance of APC/AAPC compared to zero), the terms “significant increase” or “significant decrease” were used to describe the direction of the trends.

In addition, correlations between the burden of *Clostridioides difficile* infections and the HDI were estimated by calculating the Pearson correlation coefficient. Statistical analyses were performed with the SPSS software (version 20.0, Chicago, IL, USA). For all tests, a *p* value of <0.05 was considered statistically significant.

### 2.5. Ethics Statement

The study was approved by the Ethics Committee of the Faculty of Medical Sciences, University of Kragujevac (No. 01-14321). This study was conducted using publicly available fully aggregated and anonymized data.

## 3. Results

### 3.1. Mortality Due to Clostridioides Difficile Infection

**Figure 1:**
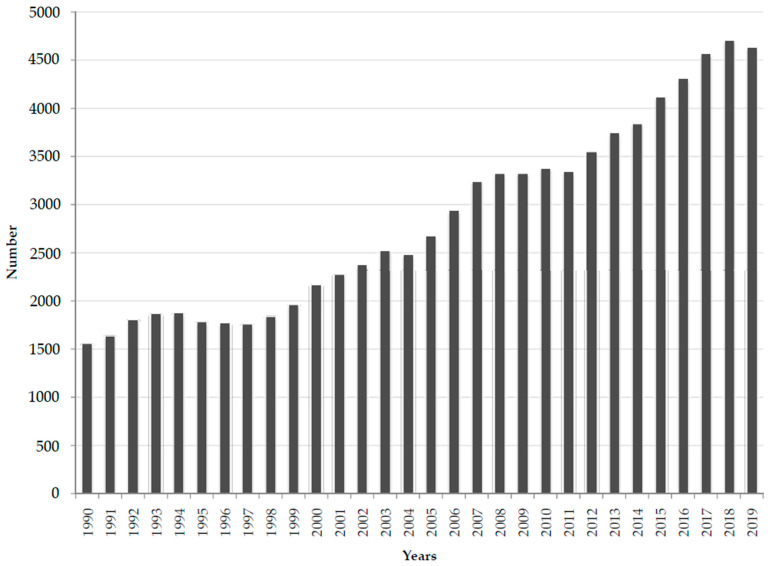
Number of deaths attributable to *Clostridioides difficile* infections in both sexes in European countries, 1990–2019. Source: Global Burden of Disease estimates [17].

**Figure 2:**
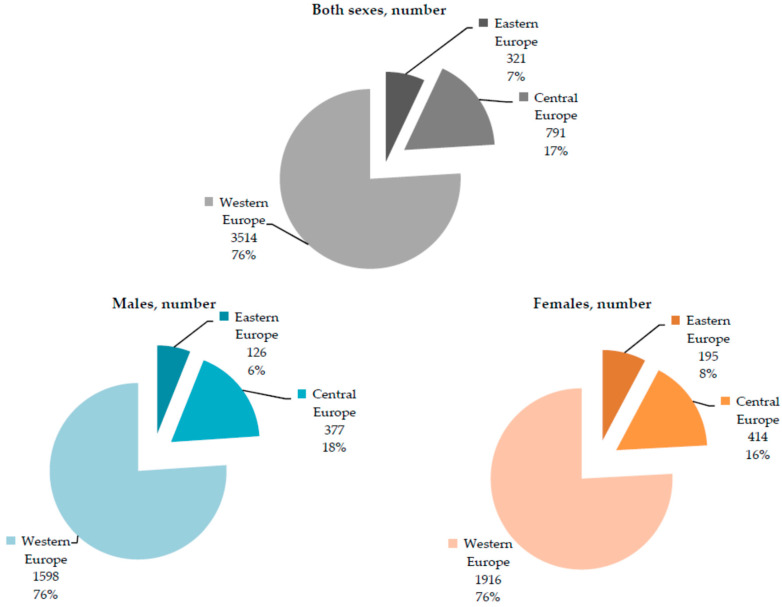
Number of deaths attributable to *Clostridioides difficile* infections in the Central, Eastern, and Western European sub-regions, by sexes, in 2019. Source: Global Burden of Disease estimates [17].

In European countries, there were about 4600 deaths attributable to *Clostridioides difficile* infections reported in 2019, with about 2100 cases (46%) in males and about 2500 cases (54%) in females (Figure 1 and Figure 2). In total, 85,300 deaths attributable to *Clostridioides difficile* infections occurred in Europe during the observed period, between 1990 and 2019, and the number of deaths showed a great increase, with the lowest number of 1500 deaths being in 1990 and the highest number of 4700 deaths being reported in 2018.

In 2019, most of the deaths attributable to *Clostridioides difficile* infections were recorded in the Western European sub-region, both in men (1598; around 76% of the total deaths in men) and women (1916; around 76% of the total deaths in women) (Figure 2). Also, in all three European sub-regions, a similar share of males (7%) and females (17%) was recorded in the total number of deaths.

**Figure 3:**
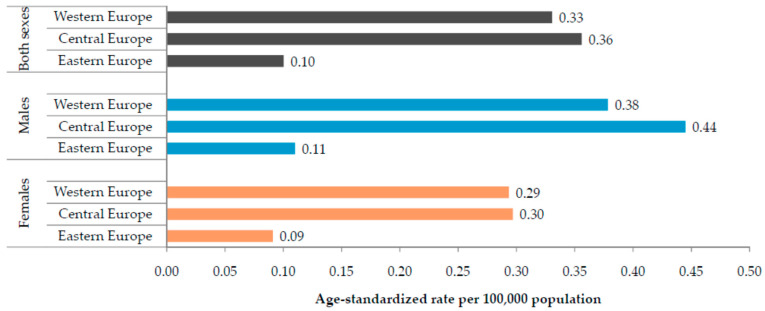
Mortality attributable to *Clostridioides difficile* infection in the Central, Eastern, and Western European sub-regions, by sexes, in 2019. Source: Global Burden of Disease estimates [17].

The age-standardized mortality rate of *Clostridioides difficile* infections in 2019 was the highest in the Central European sub-region (0.36 per 100,000), followed by the Western European sub-region (0.33 per 100,000), and the lowest rate was observed in the Eastern European sub-region (0.10 per 100,000) (Figure 3). The age-standardized YLL rates were 7.63 per 100,000 inhabitants in Central Europe, followed by the Western European sub-region (6.48 per 100,000), and the lowest rate was observed in the Eastern European sub-region (2.88 per 100,000) (Table 1). In 2019, the regional ASR of mortality due to *Clostridioides difficile* infections was equal in both sexes (i.e., 0.31 per 100,000 persons) (Table 2). The regional ASR of YLLs due to *Clostridioides difficile* infections was higher in males (6.78 per 100,000 persons) than in females (4.73 per 100,000 persons) (Table 2).

In both sexes, the highest ASRs of mortality due to *Clostridioides difficile* infections in 2019 were found in countries in the Western European sub-region: in Norway (0.93 per 100,000 persons) and Sweden (0.68 per 100,000 persons) and then in Germany (0.45 per 100,000 persons) (Table 1). The lowest ASRs (less than 0.10 per 100,000 persons) were reported in Ukraine, Belarus, Estonia, Latvia, and Montenegro.

In both sexes, the highest ASRs of YLLs due to *Clostridioides difficile* infections in 2019 were found in countries in the Western European sub-region: in Norway (16.82 per 100,000 persons) and Sweden (13.23 per 100,000 persons) and then in Germany (9.24 per 100,000 persons) (Table 1). The lowest ASRs (close to 2.00 per 100,000 persons) were reported in Ukraine, Belarus, Estonia, Latvia, and Montenegro.

### 3.2. Trends of Mortality Due to Clostridioides Difficile Infection, 1990–2019

**Figure 4:**
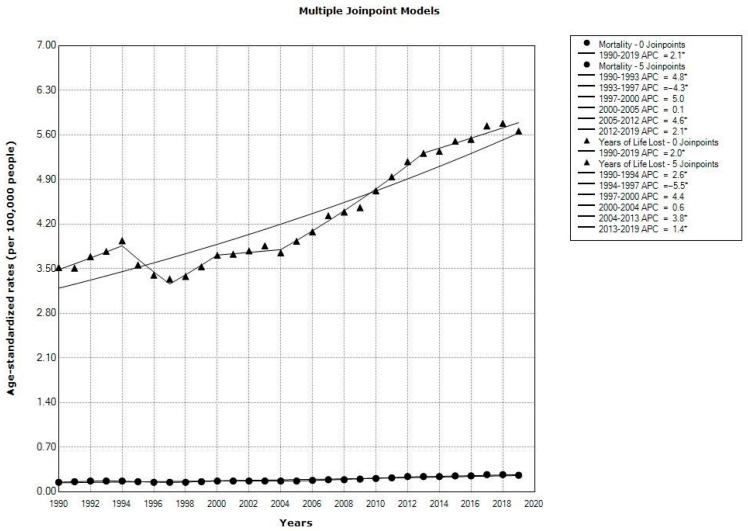
Trends in mortality and years of life lost due to *Clostridioides difficile* infection in the Central, Eastern, and Western European sub-regions as a whole, 1990–2019: a joinpoint regression analysis. * Statistically significant trend (*p* < 0.05); APC = Annual Percentage Change; AAPC, for full period presented AAPC (Average Annual Percent Change); CI = Confidence Interval. Source: Global Burden of Disease estimates [17].

Regarding the regional trends in both sexes in European countries as a whole, over the observed 1990–2019 period, the ASRs for deaths attributable to *Clostridioides difficile* infections showed a significantly increasing tendency (AAPC = +2.1%), with 5 joinpoints (mortality showed a significantly decreasing trend only from 1993 to 1997, by –4.3% per year) (Figure 4). Regarding the regional trends in both sexes in European countries as a whole, from 1990 to 2019, the ASRs for the YLLs attributable to *Clostridioides difficile* infections showed a significantly increasing tendency (AAPC = +2.0%), with 5 joinpoints (mortality showed a significantly decreasing trend only from 1994 to 1997, by −5.5% per year) (Figure 4).

A significantly increasing regional trend in mortality was recorded both in men (AAPC = +2.1%; 95% CI = 1.7 to 2.4) and women (AAPC = +2.8%; 95% CI = 2.4 to 3.2) (Table 1). Also, an increasing trend in ASRs of YLLs was recorded in men (AAPC = +1.9%; 95% CI = 1.6 to 2.2) and women (AAPC = +2.0%; 95% CI = 1.7 to 2.3) (Table 2).

When the trends in ASRs of mortality attributable to *Clostridioides difficile* infection were analyzed across the European countries, significantly increasing trends were observed in most of the countries, whereby the rise in the trend was the fastest in Italy (AAPC = +6.2%), followed by Poland (AAPC = +5.6%), Sweden (AAPC = +5.5%), and Greece (AAPC = +5.3%) (Table 1). The decreasing trends in mortality rates were recorded in several countries, whereby the decline of the trend was the fastest in Latvia (AAPC = −2.8%) and Estonia (AAPC = −2.3%).

When the trends in ASRs of YLLs attributable to *Clostridioides difficile* infection were analyzed across the European countries, significantly increasing trends were observed in most of the countries, whereby the rise in the trend was the fastest in Sweden (AAPC = +6.6%), followed by Italy (AAPC = +5.9%), and Poland (AAPC = +5.1%) (Table 1). The decreasing trends in ASRs of YLLs were observed in only 6 (out of a total of 44) countries, whereby the decline in the trend was the fastest in Ukraine (AAPC = −2.3%) and Estonia (AAPC = −2.0%).

**Table 1:** Clostridioides difficile infection: age-standardized rates (ASRs, per 100,000) of mortality and years of life lost in both sexes in the Central, Eastern, and Western European sub-regions, by location, in 1990–2019: a joinpoint regression analysis * and correlations with the Human Development Index (HDI).

Locations **	Mortality					Years of Life Lost				
	1990	2019	AAPC	Pearson Coefficient (HDI)	*p*	1990	2019	AAPC	Pearson Coefficient (HDI)	*p*
Western Europe	0.19	0.33	+2.2 *	-	-	3.72	6.48	+2.3 *	-	-
Central Europe	0.18	0.36	+3.1 *	-	-	4.05	7.63	+2.7 *	-	-
Eastern Europe	0.10	0.10	−0.6 *	-	-	2.79	2.88	−0.1	-	-
Albania	0.24	0.22	−0.6 *	−0.448	0.013	5.37	4.75	−0.8 *	−0.448	0.013
Andorra	0.10	0.09	−0.1 *	0.063	0.792	1.66	1.49	−0.4 *	−0.702	0.001
Austria	0.13	0.36	+4.9 *	0.933	<0.001	2.35	5.96	+4.6 *	0.929	<0.001
Belarus	0.08	0.07	−1.8 *	−0.796	<0.001	2.28	2.08	−0.7 *	−0.618	0.001
Belgium	0.23	0.29	+0.8 *	0.682	<0.001	4.75	6.12	+0.8 *	0.722	<0.001
Bosnia and Herzegovina	0.23	0.28	+0.7 *	−0.590	0.006	4.87	5.88	+0.8 *	0.782	<0.001
Bulgaria	0.15	0.39	−0.1 *	0.966	<0.001	3.53	8.54	+3.3 *	0.652	<0.001
Croatia	0.20	0.37	+3.8 *	0.593	0.001	4.20	7.66	+2.6 *	0.485	0.007
Cyprus	0.22	0.29	+0.8 *	0.969	<0.001	4.40	6.00	+1.0 *	0.986	<0.001
Czechia	0.27	0.39	+2.0 *	0.582	0.001	5.99	8.48	+1.7 *	0.513	0.004
Denmark	0.24	0.31	+1.0 *	0.813	<0.001	4.88	6.88	+1.3 *	0.855	<0.001
Estonia	0.09	0.06	−2.8 *	−0.920	<0.001	2.32	1.74	−2.0 *	−0.880	<0.001
Finland	0.21	0.28	+1.5 *	0.780	<0.001	3.84	5.45	+1.8 *	0.790	<0.001
France	0.22	0.28	+0.4	0.315	0.090	4.51	5.76	+0.5 *	0.392	0.032
Germany	0.29	0.45	+1.8 *	0.796	<0.001	5.96	9.24	+2.1 *	0.813	<0.001
Greece	0.06	0.22	+5.3 *	0.958	<0.001	1.06	3.32	+4.8 *	0.958	<0.001
Hungary	0.22	0.38	+2.5 *	0.595	0.001	5.13	8.50	+2.1 *	0.536	0.002
Iceland	0.14	0.20	+1.5 *	0.879	<0.001	2.50	3.70	+1.8 *	0.896	<0.001
Ireland	0.18	0.27	+1.5 *	0.820	<0.001	3.12	4.78	+1.8 *	0.828	<0.001
Israel	0.22	0.27	+0.9 *	0.747	<0.001	4.44	5.60	+0.9 *	0.750	<0.001
Italy	0.12	0.32	+6.2 *	0.766	<0.001	2.27	5.94	+5.9 *	0.738	<0.001
Latvia	0.08	0.06	−2.3 *	−0.879	<0.001	2.31	1.73	−1.7 *	−0.857	<0.001
Lithuania	0.10	0.13	−0.2	−0.165	0.383	2.46	2.99	+0.1	−0.005	0.978
Luxembourg	0.23	0.29	+0.6 *	0.496	0.005	4.71	5.82	+0.6 *	0.507	0.004
Malta	0.12	0.22	+2.4 *	0.856	<0.001	2.05	3.81	+2.7 *	0.845	<0.001
Monaco	0.11	0.17	+1.6 *	-	-	2.01	2.78	+1.3 *	-	-
Montenegro	0.05	0.05	−0.2 *	0.325	0.204	1.33	1.18	−0.5 *	−0.911	<0.001
Netherlands	0.15	0.18	+0.8 *	0.729	<0.001	2.61	3.38	+0.9 *	0.768	<0.001
North Macedonia	0.29	0.38	+0.9 *	0.960	<0.001	6.43	8.19	+0.9 *	0.949	<0.001
Norway	0.36	0.93	+4.3 *	0.956	<0.001	6.31	16.82	+4.4 *	0.952	<0.001
Poland	0.12	0.39	+5.6 *	0.774	<0.001	2.82	8.16	+5.1 *	0.756	<0.001
Portugal	0.12	0.17	+1.1 *	0.770	<0.001	2.21	3.09	+1.1 *	0.773	<0.001
Republic of Moldova	0.11	0.10	−0.7 *	−0.592	0.001	2.59	2.78	+0.3	0.277	0.138
Romania	0.20	0.38	+2.6 *	0.265	0.158	4.79	8.74	+2.2 *	0.583	0.001
Russian Federation	0.12	0.12	−0.2	−0.249	0.184	3.08	3.35	+0.3	0.198	0.294
San Marino	0.13	0.14	+0.2 *	-	-	2.20	2.23	+0.2 *	-	-
Serbia	0.20	0.24	+1.1 *	0.949	<0.001	4.26	4.94	+0.8 *	0.430	0.018
Slovakia	0.14	0.15	+0.5 *	0.945	0.005	3.29	3.21	+0.2	0.349	0.059
Slovenia	0.09	0.29	+4.4 *	0.589	0.001	2.02	5.64	+3.4 *	0.505	0.004
Spain	0.21	0.30	+1.8 *	0.930	<0.001	4.17	6.26	+2.0 *	0.841	<0.001
Sweden	0.16	0.68	+5.5 *	0.880	<0.001	2.44	13.23	+6.6 *	0.874	<0.001
Switzerland	0.23	0.29	+0.7 *	0.596	0.001	4.53	5.81	+0.9 *	0.601	<0.001
Ukraine	0.06	0.04	−3.3 *	−0.818	<0.001	2.25	1.63	−2.3 *	−0.698	<0.001
United Kingdom	0.10	0.26	+2.6 *	0.623	<0.001	1.72	4.80	+3.0 *	0.675	<0.001

* Statistically significant trend (*p* < 0.05). ** Results of correlation analysis are not shown in some locations, because there are no data of the Human Development Index in the observed period. AAPC = for full period presented Average Annual Percent Change. Source: Global Burden of Disease estimates [17].

A homogeneity of the correlation between the burden due to *Clostridioides difficile* infection and the HDI (i.e., the ASRs for the mortality and YLLs) was observed across all of the European sub-regions from 1990 to 2019. A robust (*p* < 0.001) positive correlation of ASRs for the mortality and YLLs due to *Clostridioides difficile* infection with the HDI from 1990 to 2019 was recorded in nearly all countries of the Western European sub-region and Central European sub-region, except only for France, where no significant correlation was found for mortality (*p* > 0.05) (Table 1). Contrary to this, the Pearson correlation coefficient indicated that the ASRs for mortality and YLLs due to *Clostridioides difficile* infection had a significant negative correlation with the HDI in most of the Eastern European sub-region countries.

The age-specific rates of mortality and YLLs due to *Clostridioides difficile* infection in 2019 were lower in women compared to men in all age groups (Table 2). Across all age groups, both in women and men, a significant increase in the rates of mortality and YLLs due to *Clostridioides difficile* infection was recorded. In both men and women, a significantly increasing trend in the rates of mortality and YLLs due to *Clostridioides difficile* infection was recorded across all age groups. 

**Table 2:** Trends * in rates (per 100,000) of mortality and years of life lost due to *Clostridioides difficile* infection in the Central, Eastern, and Western European sub-regions, 1990–2019: a joinpoint analysis.

	Males			Females		
Age	Age-Specific Rates		AAPC (95% CI)	Age-Specific Rates		AAPC (95% CI)
	1990	2019		1990	2019	
	Mortality					
<5	0.06	0.07	+0.9 * (0.7 to 1.2)	0.03	0.04	+0.8 * (0.7 to 1.0)
5–9	0.02	0.03	+0.9 * (0.6 to 1.2)	0.03	0.03	+1.3 * (1.0 to 1.6)
10–14	0.01	0.02	+1.0 * (0.8 to 1.3)	0.02	0.03	+1.4 * (1.0 to 1.7)
15–19	0.01	0.02	+1.3 * (1.1 to 1.5)	0.01	0.02	+2.1 * (1.7 to 2.4)
20–24	0.01	0.02	+1.4 * (1.1 to 1.7)	0.01	0.02	+2.2 * (1.8 to 2.5)
25–29	0.02	0.03	+1.8 * (1.4 to 2.2)	0.01	0.02	+2.1 * (1.8 to 2.5)
30–34	0.03	0.05	+2.0 * (1.6 to 2.5)	0.02	0.03	+2.5 * (2.1 to 2.9)
35–39	0.04	0.06	+2.0 * (1.6 to 2.4)	0.02	0.04	+2.4 * (2.0 to 2.8)
40–44	0.05	0.08	+2.0 * (1.6 to 2.3)	0.03	0.05	+2.3 * (2.0 to 2.6)
45–49	0.07	0.11	+2.0 * (1.7 to 2.2)	0.04	0.07	+2.5 * (2.2 to 2.8)
50–54	0.10	0.15	+1.9 * (1.6 to 2.2)	0.05	0.09	+2.1 * (1.8 to 2.4)
55–59	0.13	0.18	+1.6 * (1.4 to 1.9)	0.07	0.11	+1.9 * (1.6 to 2.1)
60–64	0.25	0.41	+2.1 * (1.8 to 2.4)	0.16	0.25	+2.1 * (1.8 to 2.4)
65–69	0.50	0.95	+2.6 * (2.2 to 3.0)	0.34	0.62	+2.6 * (2.2 to 3.0)
70–74	0.85	1.66	+2.6 * (2.2 to 3.0)	0.61	1.14	+2.4 * (2.1 to 2.8)
75–79	1.46	2.62	+2.4 * (1.9 to 2.8)	1.14	1.93	+2.1 * (1.7 to 2.5)
80–84	2.51	4.45	+2.2 * (1.7 to 2.6)	2.04	3.47	+2.0 * (1.7 to 2.4)
85–89	4.56	7.64	+2.0 * (1.5 to 2.4)	3.64	6.05	+2.0 * (1.6 to 2.3)
90–94	7.62	11.66	+1.6 * (1.1 to 2.0)	6.08	9.62	+1.8 * (1.5 to 2.2)
95+	11.08	16.64	+1.4 * (1.1 to 1.7)	9.40	13.86	+1.6 * (1.2 to 2.0)
	Age-standardized rates					
All ages	0.19	0.31	+2.1 * (1.7 to 2.4)	0.15	0.31	+2.8 * (2.4 to 3.2)
	Years of Life Lost					
<5	5.20	6.35	+0.9 * (0.7 to 1.1)	2.67	3.23	+0.8 * (0.7 to 1.0)
5–9	2.01	2.40	+0.9 * (0.6 to 1.1)	2.07	2.59	+1.3 * (1.0 to 1.6)
10–14	0.96	1.18	+1.1 * (0.8 to 1.3)	1.37	1.86	+1.4 * (1.1 to 1.8)
15–19	0.95	1.24	+1.3 * (1.1 to 1.5)	0.74	1.14	+2.0 * (1.7 to 2.3)
20–24	1.00	1.36	+1.5 * (1.2 to 1.7)	0.54	0.85	+2.0 * (1.7 to 2.4)
25–29	1.33	1.97	+1.8 * (1.4 to 2.2)	0.78	1.23	+2.0 * (1.7 to 2.4)
30–34	2.01	2.40	+0.9 * (0.6 to 1.1)	0.87	1.55	+2.5 * (2.1 to 2.8)
35–39	2.07	3.32	+2.0 * (1.6 to 2.4)	1.20	2.05	+2.4 * (2.0 to 2.7)
40–44	2.49	4.03	+1.9 * (1.6 to 2.3)	1.44	2.43	+2.3 * (2.0 to 2.6)
45–49	3.17	4.89	+2.0 * (1.7 to 2.2)	1.63	2.90	+2.5 * (2.2 to 2.8)
50–54	3.73	5.61	+1.9 * (1.6 to 2.2)	2.05	3.41	+2.1 * (1.8 to 2.4)
55–59	4.17	6.16	+1.6 * (1.4 to 1.9)	2.41	3.67	+1.8 * (1.6 to 2.1)
60–64	7.20	11.90	+2.1 * (1.8 to 2.4)	4.56	7.23	+2.1 * (1.8 to 2.4)
65–69	12.21	23.14	+2.6 * (2.2 to 3.0)	8.33	15.14	+2.6 * (2.2 to 3.0)
70–74	17.03	33.31	+2.6 * (2.2 to 3.0)	12.10	22.80	+2.4 * (2.0 to 2.8)
75–79	23.25	41.52	+2.3 * (1.9 to 2.8)	18.12	30.51	+2.1 * (1.7 to 2.4)
80–84	31.51	55.47	+2.2 * (1.7 to 2.6)	25.48	42.98	+2.0 * (1.7 to 2.3)
85–89	45.48	75.54	+2.0 * (1.5 to 2.4)	36.06	59.50	+2.0 * (1.7 to 2.3)
90–94	65.73	100.47	+1.6 * (1.1 to 2.0)	52.44	82.82	+1.8 * (1.4 to 2.1)
95+	90.69	134.19	+1.3 * (1.0 to 1.7)	76.28	111.98	+1.6 * (1.2 to 2.0)
	Age-standardized rates					
All ages	4.26	6.78	+1.9 * (1.6 to 2.2)	2.95	4.73	+2.0 * (1.7 to 2.3)

* Statistically significant trend (*p* < 0.05); AAPC, for full period presented AAPC (Average Annual Percent Change); CI = Confidence Interval. Source: Global Burden of Disease estimates [17].

## 4. Discussion

This study indicates a rising burden of disease attributable to *Clostridioides difficile* infection in most of the European countries in the last decades. The significantly increasing trends in the mortality and YLLs due to *Clostridioides difficile* infection were positively correlated with the level of the country’s development.

The trend of the regional burden due to *Clostridioides difficile* infection showed a significant increase over the 1990–2019 period, and this was recorded in nearly all countries, among both sexes and all analyzed age groups. Similar patterns of *Clostridioides difficile* infection mortality were reported in some previous studies, primarily in countries in North America [9,14,24]. In the United States of America, *Clostridioides difficile* infection was responsible for about half a million infections and 29 000 deaths in 2012, whereby close to two thirds of these cases were associated with hospitalizations, and over 80% of the deaths were recorded in elderly patients [9]. The rise in the burden of *Clostridioides difficile* infection in the world over the last few decades could be associated with the growth and aging of the population and individuals in some of the recognized population groups that are at a high risk, including the elderly, hospitalized patients, or those under antimicrobial therapy [25,26,27]. Although primarily associated with the healthcare setting, *Clostridioides difficile* infection has recently been recorded increasingly in the community setting in persons without a connection to a healthcare system or exposure to antibiotics [9,14,15,28,29]. Additionally, socio-economic developments could at least in part be the reason behind the observed great international differences, both between regions and countries and in the direction and magnitude of the trends in mortality rates attributable to *Clostridioides difficile* infection. Recently, it was reported that most of the countries that experienced the highest burden attributable to *Clostridioides difficile* infection belonged to the high socio-demographic index areas (a composite measure of the averages of incomes per capita, educational attainments, and fertility rates), with the burden being relatively low in developing areas [28]. Inequities in healthcare infrastructure availability and development could be related to differences in the geographical distribution and frequency of *Clostridioides difficile* infection worldwide, because in countries with limited resources, preventive/diagnostic/therapeutic resources are allocated elsewhere, and patients with diarrhea (community- and nosocomial-acquired) might not be fully assessed. Also, the issue of a possible under-representation and under-reporting of *Clostridioides difficile* infections owing to differences in surveillance is present worldwide, particularly in limited-resource countries [30,31]. Furthermore, most *Clostridioides difficile* infections are without symptoms; therefore, it is reasonable to consider the extent to which these help in spreading this infection in different areas [32,33].

Finally, a lack of effective specific preventive measures against *Clostridioides difficile* infection is of great relevance for inopportune international incidences and mortality trends [34]. Also, many reports indicate that, in recent years, *Clostridioides difficile* infection (ribotypes 027 and 078) has spread to new areas, including Europe, where the presence of the local transmission and occurrence of several epidemics was recorded [34,35].

The findings of this research are in line with reports of some authors who noted that age-standardized mortality rates are somewhat higher in males than females; however, these reports were not consistent [1]. The discrepancies in burden by gender could be due to biological discrepancies that are sex-related and that are exposure and socio-economic differences. These discrepancies in the mortality burden by sex might, at least to some extent, be due to some innate sex-related hormonal and genetic dissimilarities.

The worldwide observed increase in the trend of burden attributable to *Clostridioides difficile* infection implies that this disease poses a public health threat globally and requires more effective measures of prevention to be developed (i.e., specific treatment interventions and developments of vaccines). Ongoing research efforts are focused on exploring newer antibiotics [36] and identifying potential candidates for vaccines against *Clostridioides difficile* infections (i.e., toxins, cell–surface components, and spore proteins) [37].

Furthermore, the burden attributable to *Clostridioides difficile* infection can be reduced by improving healthcare availability and access and improving socio-economic statuses around the world. Information about disease burdens is essential for healthcare policymakers to appropriately allocate resources that are limited. The COVID-19 pandemic led to an overloading of health facilities, and many various health services were performed with a reduced capacity or were suspended, such as registries, autopsies, surveys, etc. Future analytical epidemiologic studies could provide an explanation for significant differences in the burden trends of *Clostridioides difficile* infection in European countries.

### Strengths and Limitations of This Study

According to the data from the available literature, this study is the first to present the trends in burden of *Clostridioides difficile* infection in European countries in the last three decades. However, several sources of limitation should be considered in this study. First, although the findings of this study are based on the GBD data as one of the most comprehensive disease burden assessments worldwide, the question of data accuracy and reliability can always be raised. Consequently, a limitation specific to evaluating deaths related to *Clostridioides difficile* infection can lead to mortality under-estimations with regard to how developed and available healthcare services are across countries. On the other hand, the technological advances in some of the most developed countries may have enabled a more sensitive/specific diagnostics in the studied period, which can partly be linked to the observed trend of increased mortalities from Clostridioides difficile infections. Further, since the GBD study data were publicly available until 2019, the potential effect of the COVID-19 pandemic on mortalities from *Clostridioides difficile* infections in European countries was not evaluated in this study. Further efforts are needed in providing reliable data as an important factor in the prevention and control of this disease. Finally, fallacies inherent to the ecological study design that was applied in this research are potential limitations of this study. However, despite these limitations, this study provides useful insights into the geographical and temporal variations in the burden of disease attributable to *Clostridioides difficile* infections in European countries and could help healthcare decision makers in creating more successful strategies for disease prevention on the basis of reliable disease burden estimates.

## 5. Conclusions

There are large differences in the burden of *Clostridioides difficile* infection across European countries. The highest burden and increasing trends of the burden of *Clostridioides difficile* infection were seen in most of the developed countries in the Western European sub-region and Central European sub-region. Directions for future research should involve factors that could have contributed to great geographic variations in the burden of *Clostridioides difficile* infection to enable determining measures for a further control and management of this disease that would be more effective.

## Data Availability

Data are contained within the article.
